# Epidemiological Research Advances in Vascular Calcification in Diabetes

**DOI:** 10.1155/2021/4461311

**Published:** 2021-10-01

**Authors:** Haipeng Yao, Zhen Sun, Guangyao Zang, Lili Zhang, Lina Hou, Chen Shao, Zhongqun Wang

**Affiliations:** Department of Cardiology, Affiliated Hospital of Jiangsu University, Zhenjiang, China

## Abstract

Vascular calcification is the transformation of arterial wall mesenchymal cells, particularly smooth muscle cells (SMCs), into osteoblast phenotypes by various pathological factors. Additionally, vascular transformation mediates the abnormal deposition of calcium salts in the vascular wall, such as intimal and media calcification. Various pathological types have been described, such as calcification and valve calcification. The incidence of vascular calcification in patients with diabetes is much higher than that in nondiabetic patients, representing a critical cause of cardiovascular events in patients with diabetes. Because basic research on the clinical transformation of vascular calcification has yet to be conducted, this study systematically expounds on the risk factors for vascular calcification, vascular bed differences, sex differences, ethnic differences, diagnosis, severity assessments, and treatments to facilitate the identification of a new entry point for basic research and subsequent clinical transformation regarding vascular calcification and corresponding clinical evaluation strategies.

## 1. Introduction

The global prevalence of diabetes is currently rapidly increasing. According to data released by the International Diabetes Federation (IDF), in 2019, 463 million adults aged 20–79 years worldwide had diabetes (9.3% of all adults in this age group). By 2030, estimates indicate that 578.4 million people will have diabetes; by 2045, 702 million adults are expected to have diabetes. Vascular calcification is a common complication in patients with diabetes and a critical cause of cardiovascular disease and death. However, the mechanism underlying vascular calcification in patients with diabetes remains unclear. In recent years, vascular calcification was identified as a complex, active, and highly adjustable pathophysiological process mediated by various cells. Vascular calcification is characterized by the transformation of vascular cells from their original contractile phenotype into an osteoblast-like phenotype following stimulation with various factors [1, 2]. Vascular smooth muscle cells (VSMCs) are the primary cells responsible for vascular calcification. The degradation products of smooth muscle cells after apoptosis or necrosis (apoptotic bodies and stromal vesicles, respectively) are involved in the initiation of VSMC calcification [2]. When vascular smooth muscle cells are exposed to stimuli, such as oxidative stress and high-glucose levels, they differentiate into osteoblast-like cells and secrete large amounts of bone-related proteins. Examples include alkaline phosphatase (ALP), core-binding factor a1 (Cbfa1), osteocalcin (OCN), type I collagen (Col I), and bone morphogenetic protein 2 (BMP-2). These proteins facilitate the deposition of calcium phosphate in the extracellular matrix and the occurrence of vascular calcification [3–6]. Additionally, many dynamic changes also trigger or promote vascular calcification. For example, matrix Gla protein (MGP), inorganic pyrophosphate, osteoprotegerin, osteopontin (OPN), fetuin A (FA), and other vascular calcification inhibitors are lost, or high blood sugar levels, reactive oxygen species (ROS), inflammatory cytokines, G protein-coupled receptors- (GPCR-) *β* 2-Ar signaling pathway, AGE and its receptors (RAGE), which constitute the AGE/RAGE signaling pathway, and other cartilage factors are induced or enhanced [7–11]. Although these factors explain the pathogenesis of vascular calcification from several different aspects, many links between basic science and clinical transformation have not been elucidated. This study systematically discusses the clinical aspects of vascular calcification risk factors, vascular bed differences, sex differences, and ethnic differences, along with the diagnosis, severity assessments, and treatment options to identify a new entry point for the basic and clinical transformation of vascular calcification and corresponding clinical evaluation strategies.

## 2. Risk Factors for Vascular Calcification in Patients with Diabetes

### 2.1. High Blood Sugar Levels

In recent years, many studies have investigated the mechanism by which hyperglycemia promotes vascular calcification. VSMCs cultured under high-glucose conditions express RUNX2, BMP-2, and OCN at high levels and exhibit enhanced matrix calcification [12]. In a study using mice [13], high glucose promoted the transformation of mouse VSMCs into osteoblasts with high expression of decorin (DCN). DCN is an essential component of the extracellular mechanism. Exogenous DCN inhibits the activity of extracellular matrix-degrading enzymes and collagen degradation while increasing matrix deposition and calcification. Similarly, in vitro studies have found that high-glucose levels induce the calcification of VSMCs [5]. When vascular smooth muscle cells undergo oxidative stress following exposure to high-glucose levels and other stimulating factors, they differentiate into osteoblast-like cells and secrete large amounts of bone-related proteins. Kawakami and colleagues [14] found that the S100 family is closely related to vascular calcification. Hyperglycemia promotes increased S100A9 secretion and increased expression of RAGE protein receptors. In a high-glucose environment, proinflammatory macrophages release calcified extracellular vesicles (EVs), which form atherosclerotic microcalcifications through the S100A9-RAGE axis [15]. High-glucose-dependent mitochondrial superoxide production becomes excessive, leading to NF-*κ*B activation [16], and NF-*κ*B induces S100A9 expression. The interaction of S100A9 and RAGE activates the NF-*κ*B pathway, resulting in a positive feedback loop. S100A9 induces the secretion of proinflammatory cytokines through mechanisms such as ROS generation and activation of ROS-sensitive transcription factors (NF-*κ*B). Hyperglycemia also promotes increased expression of BMP-2/4 proteins. In patients with diabetes, BMP-2/4 expression in the aorta increases [17]. The protein induces the osteogenic differentiation of VSMCs, leading to cellular calcification.

### 2.2. Lipid Metabolism Disorder

A high-fat diet can lead to obesity, high blood pressure, diabetes, and vascular calcification. In the high-glucose state of diabetes, oxidized low-density lipoprotein further promotes atherosclerosis and calcification of the vascular media. Previous studies have suggested that HDL cholesterol exerts an antiarteriosclerotic effect, and related in vitro cell-based experiments have shown that HDL cholesterol lacking oxidative modifications inhibits vascular calcification [18]. Decreased HDL cholesterol levels are often accompanied by increased leptin levels in blood circulation. Increased leptin levels upregulate the BMP osteogenic signaling pathway, stimulate the differentiation of VSMCs into osteoblasts, and mediate the formation of vascular calcification [19]. Proudfoot and colleagues [20] found that when dyslipidemia occurred, acetylated low-density lipoprotein (LDL) increased the osteogenic phenotype of VSMCs by 3-fold. Thus, LDL induces VSMC differentiation and increases ALP activity. In contrast, HDL inhibits the osteogenic differentiation pathway [21]. Additionally, hypercholesterolemia increases oxidative stress and accelerates VD-induced vascular calcification [22]. Bjornstad and colleagues [23] found that triglycerides independently predict the progression of coronary artery calcification (CAC) in patients with diabetes. Some studies have found that hyperlipidemia is related to Wnt/*β*-catenin signaling, which plays a vital role in vascular calcification [24].

### 2.3. Insulin Abnormalities

Insulin exerts a protective effect on arterial vascular calcification. Numerous studies have shown that NO inhibits platelet activation and limits the migration and proliferation of VSMCs [25]. Insulin stimulates the vascular endothelium to release NO that subsequently oxidizes lipoproteins, thereby reducing the rate of intimal calcification and inhibiting the formation of VSMCs [26]. When insulin resistance (IR) occurs, more free fatty acids enter the liver, and the body compensates by increasing the hepatic absorption of triglycerides and the production and secretion of VLDL; thus, the risk of vascular calcification also increases. As shown in the study by Iguchi and colleagues, patients with higher levels of IR are more likely to develop thin cap fibrous atherosclerosis (TCFA), and the cap is significantly thinner [27]. IR promotes atherosclerosis by inducing the inflammatory activity of blood vessels and immune cells. Additionally, the higher the IR index is, the higher the CAC is [28]. However, another study conducted in 1632 nondiabetic people showed that IR is only related to CAC and not to abdominal aortic calcification or thoracic aortic calcification [29]. Additionally, hyperinsulinemia decreases OPG and MGP levels, activates BMP-2, and increases CBFA-1 and ALP levels, leading to the bone-like transformation of interstitial cells. When hyperinsulinemia occurs, the clearance of Kupffer cells in the liver decreases [30]. This change leads to decreased clearance of lipopolysaccharides absorbed from the gastrointestinal tract and a corresponding increase in circulating lipopolysaccharide and insulin levels. Additionally, in a rat study, inhibition of Kupffer cells caused increased glucose levels in the body and stimulated excessive insulin secretion, leading to IR [31]. Stefan and colleagues [32] found that plasma globulin A inhibits insulin signal transduction and induces IR, leading to atherosclerosis.

### 2.4. Obesity

Obesity is an independent risk factor for the development of cardiovascular disease. Obesity is common in patients with T2DM, while the average obesity rate in patients with T1DM is not higher than that of the general population. In patients with type 1 diabetes, obesity is closely related to the existence and progression of CAC. Currently, the relationship between obesity and T2DM is unclear. In a British study on all-cause mortality in patients with T2DM, patients with a BMI of 35–54 kg/m^2^ or 20–24 kg/m^2^ had an increased risk of all-cause mortality [33]. However, in a study of subclinical atherosclerosis in 52 overweight and obese patients with T2DM, obesity showed a weaker correlation with CAC [34]. Therefore, obesity may be less of a risk factor for vascular calcification in patients with diabetes. Studies have shown that epicardial fat is related to CAC, and the interaction between epicardial fat tissue and the coronary artery occurs through paracrine activity involving inflammatory markers, which may mediate and induce calcification [35]. In a study of 1414 African Americans, CAC and AAC were associated with epicardial fat [36]. Katsiki et al. also confirmed that epicardial fat is related to risk factors for vascular calcification [37].

### 2.5. Hypertension

The renin-angiotensin-aldosterone system is a major pathogenic factor contributing to VSMC apoptosis, growth, and differentiation, thus suggesting the possible involvement of the system in arterial calcification. Ang II promotes VSMC differentiation into osteoblasts by activating RANKL [38]. Similarly, once aldosterone (Aldo) is overactivated, it promotes the progression of cardiovascular disease events. When Aldo is abnormally activated, it exerts a negative effect on blood vessel function and promotes vascular stress, proliferation, and vascular inflammation [39]. Studies have also shown that the promoter sequence of the pit-1 gene may contain an MRE, and Aldo triggers osteoinduction through pit-1. Pit-1 regulates phosphate absorption and is essential for phenotypic transformation and vascular calcification of VSMCs [40]. Li and colleagues [41] revealed that silencing pit-1 with a small interfering RNA reduced the mRNA levels of Cbfa1 and ALP and inhibited vascular calcification. Although many studies have investigated the role of aldosterone in vascular calcification, these findings provide a new target to treat vascular calcification (VC) [42]. The contribution of hypertension to VC remains unclear.

### 2.6. Kidney Disease

Epidemiological surveys have shown that people with diabetes are approximately twice as likely to develop chronic kidney disease as those without diabetes. A study of calcification in Chinese patients undergoing dialysis shows that the overall calcification rate in patients receiving dialysis is 77.4%. The incidence of CAC in nondialysis patients with type 2 diabetes presenting renal damage was higher than that in patients with type 2 diabetes without renal damage (95% and 59%), and the median CAC score was significantly higher [43]. With the continuous deterioration of renal function in patients with chronic kidney disease, the probability of calcium and phosphorus metabolism disorder increases, and the degree of vascular calcification will be significantly increased. The microinflammatory state plays a key role in promoting the occurrence and development of vascular calcification. The microinflammatory state is a nondominant inflammatory state caused by an infection with a nonpathogenic microorganism and is characterized by low levels and continuous increases in inflammatory proteins and inflammatory cytokines in the systemic circulation and is common in patients with chronic kidney disease. A slight increase in these indexes is positively correlated with calcification-promoting factors, indicating that the microinflammatory state plays a role in promoting calcification.

### 2.7. Aging

Vascular calcification is a sign of aging. According to recent studies, aging promotes vascular calcification by inducing the osteogenic transformation of vascular smooth muscle cells, release of vesicles by endothelial cells, remodeling of extracellular matrix, imbalance of phosphorus metabolism, DNA damage, inflammatory response, and reduced expression of antiaging factors [44–46]. At the same time, age also reflects the cumulative exposure of arterial media to other influencing factors. For example, advanced glycation end products (AGEs) are the end products of nonenzymatic glycosylation reactions. They are proteins that are generated and accumulate in tissues with age. In particular, the AGE concentration in elderly patients with type 2 diabetes is significantly higher than that in nondiabetic patients [47]. During hyperglycemia, AGE/RAGE signals are transmitted through PKC, p38 MAPK, TGF-*β*, and NF-*κ*B, and other signaling pathways increase bone matrix proteins [48–50]. Moreover, AGE/RAGE signaling has been shown to increase oxidative stress and promote diabetes-mediated vascular calcification by activating Nox-1 and decreasing SOD-1 expression [51, 52]. In addition, the risk of lower extremity arterial disease (LEAD) in patients with diabetes and a history of more than 20 years of drinking was significantly higher than that in nondrinkers [53], suggesting that the relationship between alcohol consumption and vascular calcification also indicates that age plays an important role in vascular disease.

## 3. Distribution of Different Vascular Beds

Vascular calcification occurs in the intima and media. Intimal calcification occurs in cerebral arteries, carotid arteries, coronary arteries, aorta, renal arteries, and peripheral arteries of the limbs. Media calcification often coexists with intimal calcification but also exists independently of intimal calcification. In particular, it is common in lower extremity arterial disease in patients with diabetes [54]. When observed using ultrasound, the media and intima are distinct. Ultrasound reveals that calcification of the arterial media is continuous, smooth, and linearly hyperechoic in the blood vessel wall. Intimal calcification manifests as patchy protrusions from the lumen, disconnected calcification, and irregular hyperechoic calcification [55].

Differences in vascular calcification in different vascular beds have also been reported in recent years. One of the most studied types is CAC, which is a critical predictor of cardiovascular events independent of traditional risk factors. Similarly, the presence of thoracic aortic calcification is independently associated with total mortality [56], and the presence of abdominal aortic calcification is associated with fatal and nonfatal cardiovascular events [57]. Allison and colleagues [58] found a difference in the prevalence of calcification among different vascular beds in 4544 patients who had undergone whole-body CT scans during an 8-year follow-up. Among them, calcification in the coronary artery had the highest prevalence (55.8%), followed by the abdominal aorta (54.8%), while calcification in the carotid artery had the lowest prevalence rate (32.2%). In a multiethnic study of atherosclerosis (MESA), the association between abdominal aortic calcification (AAC) and diabetes was the strongest among all vascular beds [59]. Patients with calcification in the carotid, coronary, and iliac arteries have a significantly higher body mass index, and patients with calcification in any vascular bed other than the carotid artery have a higher probability of a family history of cardiovascular disease [58]. Cox and colleagues [60] found that for all-cause mortality, each increase in the standard deviation of CAC was associated with an approximately 1.8-fold increase in risk, while risks of CarCP and AACP increased by approximately 1.5-fold and 1.4-fold, respectively. This trend was also observed for the risk of cardiovascular disease mortality. However, in a study of 4,544 elderly individuals (average age: 56.8 years), Allison and colleagues [58] found that the abdominal aorta was associated with the highest all-cause mortality and CVD mortality among all vascular beds. Compared with changes in the coronary arteries, carotid arteries, and iliac arteries, the changes detected in the thoracic aorta are associated with a more than 2-fold increase in all-cause mortality, and this correlation is smallest in the coronary arteries. These different results may be attributed to the different prevalence rates of T2DM among study subjects and the low prevalence of early vascular calcification.

## 4. Sex Differences in Vascular Calcification

Many studies have shown sex differences in vascular calcification. Kronmal and colleagues found that the male sex was a significant risk factor for CAC. Compared with women, the prevalence of CAC in men is 43% higher [61]. The first appearance of atherosclerosis in women occurs 10 years later than in men [62]. Allison and colleagues evaluated 650 asymptomatic subjects and found that 47% of women younger than 50 years had calcifications compared with 70% of men [63]. This difference persists in the 50- to 60-year-old and 60- to 70-year-old age groups. However, after females undergo menopause, the prevalence of vascular calcification in women becomes significantly higher than that in men. This finding suggests that hormones related to menopause in females, such as estrogen, may exert a protective effect on the development of vascular calcification. However, studies have also shown that estrogen does not exert a protective effect on the more common plaque erosion in women [64].

The size of the sex difference in vascular calcification varies between races [65]. White men have the highest prevalence of CAC. However, another study of 16,560 asymptomatic subjects found no significant sex difference in vascular bed calcification between Hispanic and Caucasian subjects [66].

Currently, the explanation for the racial/ethnic differences in CAC sex differences remains speculative. Vitamin D gene polymorphisms are related to CAC levels [67]. Sex differences were found in vitamin D polymorphisms, and the size of the difference varied with ethnicity [65]. This finding suggests that vitamin D may be a mediating factor of sex differences in CAC. Among other vascular beds, white and Hispanic men are more likely to have aortic calcification (AVC) than women. Regarding the prevalence of thoracic aortic calcification (TAC), this sex difference seems to be reversed.

Except for African Americans, men of other races negatively correlate with the prevalence of TAC [68]. Niccoli et al. found that women have a higher prevalence of TAC than men [69]. Regarding abdominal calcification, Hess and Grant found that women have a higher incidence of abdominal calcification [70]. Additionally, sex differences were found in the earliest calcifications in the vascular bed. Men and women show the earliest calcifications in the coronary arteries and distal aorta, respectively.

## 5. Ethnic Differences in Vascular Calcification in Patients with Diabetes

In recent years, an increasing number of studies have identified significant ethnic differences in the prevalence of vascular calcification (Table 1). Among African Americans, each increase in standard deviation of individuals of European descent was associated with an 8% increase in the prevalence of coronary calcification [71]. However, another study did not appear to prove this result. Villadsen et al. conducted a study involving 420 Caucasian men and 543 men from South Asia, and reported that the Agatston calcium score in the diabetic group was significantly higher than that in the black group, but in the nondiabetic group, the difference was not confirmed [72]. Another study also confirmed that the CAC of white individuals is significantly higher than that of black individuals. Even after adjusting for CVD risk factors, this difference persisted [73]. Bild and colleagues [74] conducted the MESA study and found that compared with white individuals, the relative risks for coronary calcification were 0.78 (95% CI: 0.74–0.82) in black individuals, 0.85 (95% CI: 0.79–0.91) in Hispanic individuals, and 0.92 (95% CI 0.85–0.99) in Chinese individuals. Although the prevalence of CAC in individuals of African descent is lower than that in Caucasians, the incidence and mortality of coronary heart disease in individuals of African descent are higher than that of Caucasians [75]. Matthew and others [66] observed a significantly higher prevalence of CAC in Caucasians and Hispanics than in African and Asian men. After adjusting for related risk factors that increase the presence and severity of atherosclerosis because of the occurrence of kidney disease, these differences persisted, whereas the effect of diabetes ranged from 105% for more severe calcification (CAC > 400) in Caucasians to 236% for more severe calcification in Asians. This conclusion confirms that vascular calcification caused by diabetes exerts a greater effect on Asians. In addition, compared with Japanese men, Caucasian American men had a heavier burden of coronary atherosclerosis, although Japanese men had higher blood pressure, total cholesterol levels, low-density lipoprotein levels, and smoking rates. Moreover, ethnic differences also increase with age, especially among people aged 45–74 years [76]. Another very interesting phenomenon is that the mortality rate of ASCVD (atherosclerotic cardiovascular disease) in South Asians (people from Bangladesh, India, Bhutan, the Maldives, Nepal, Pakistan, and Sri Lanka) is higher than that in other Asian groups and Caucasians, which is in sharp contrast to the finding that Asian Americans have a lower risk of ASCVD as a group [77]. The greatest risk factor contributing to this racial difference is the high incidence rate of diabetes and impaired glucose tolerance in South Asians. The Agatston score of Caucasians is also slightly higher than that of South Asians (SA), and the burden of calcified plaques is also heavier, but the CAC scores of Germans and Caucasians are similar [78].

Intra-arterial media thickness (IMT) is also a reliable indicator of atherosclerosis and can be used to quantify the burden of atherosclerotic disease in different ethnic groups. Compared with white individuals, black individuals usually have a thicker common carotid artery IMT and thinner internal carotid artery IMT. Using ultrasound to evaluate internal carotid artery atherosclerosis and predict subsequent coronary events, we found that black individuals had the highest common carotid artery IMT, but the internal carotid artery IMT was similar to that of white and Spanish individuals, and Chinese individuals had the lowest carotid IMT, particularly with respect to the internal carotid artery. Ethnic differences exist in the relationship between coronary artery calcification and carotid IMT [79]. For every 10% increase in the CAC score, the adjusted increase in internal carotid artery IMT was highest among Hispanic individuals (approximately 18.5%) and lowest among black individuals (approximately 6.1%). The prevalence and score of internal carotid artery IMT and CAC increased significantly with age [74]. Among these populations, Spanish men exhibited the smallest increase in coronary calcification with age, while white individuals exhibited the largest increase in IMT and calcification scores. Regardless of race, the common carotid artery IMT in patients with CAC is greater than that in patients without CAC. Regardless of whether CAC occurs, the greatest thickness of the carotid artery intima is observed in black individuals.

Wong and colleagues [80] reported that the prevalence of TAC ranges from 10% in men without CAC to 64% in men with CAC and from 15% to 82% in women (*P* < 0.0001). Although the incidence of TAC among Chinese, black, and Hispanic individuals is lower than that among white individuals, only black individuals have a significantly lower relative risk of TAC than white individuals. In the multivariate model, the TAC progression of Hispanic individuals was significantly lower than that of white individuals by 14.8 units and that of black individuals was lower than that of white individuals by 18.4 units [81]. However, a cross-sectional survey of elderly hemodialysis patients with a high prevalence of diabetes in the United States showed no difference in the prevalence of coronary and thoracic aortic calcification between white and African American individuals [82]. Other studies also indicate no difference in the prevalence of coronary artery calcification between black and non-Hispanic white individuals [83].

In conclusion, compared with white individuals, the prevalence and severity of CAC and TAC in African American and Asian individuals are much lower, but the incidence and mortality of coronary heart disease in black individuals are higher. The reasons for ethnic differences might be related to differences in calcium metabolism [84], VD metabolism, and genetic and environmental factors. However, the specific reasons for this difference require further study.

## 6. Diagnosis and Severity Assessments

Patients with T2DM are at increased risk of adverse atherosclerotic events, including acute coronary syndrome (ACS) and ischemic stroke [85]. Early identification and prediction of severity are critical. Previously, various risk assessment algorithms, such as the Framingham risk score (FRS) and systematic coronary artery risk assessment (SCORE), were used to predict the likelihood of cardiovascular events [86]. Although these algorithms are considered useful tools for risk stratification in the general population, they lack sufficient capabilities and accuracy in patients with T2DM [87]. Currently, they are surrogate indicators for evaluating the risk of arterial calcification. Brachial artery flow-mediated dilation (FMD), carotid atherosclerotic burden, ankle-brachial index (ABI), arterial stiffness, CT coronary artery calcification scores (CACs), and invasive imaging testing have been increasingly used to predict cardiovascular events [88–90].

### 6.1. Invasive Imaging Testing

Invasive imaging has become increasingly widely used in the detection of arterial calcification. As the clinical gold standard for evaluating coronary atherosclerosis disease, coronary angiography (CAG) is used to diagnose and quantify the degree of stenosis of the arterial lumen and has a high specificity. However, compared to other invasive imaging methods, the sensitivity (40%) is relatively low [91]. With the continuous increase in CAC, the lesions involve the vascular wall, preventing clinicians from accurately evaluating the imaging data [91].

Intravascular ultrasound (IVUS) involves an imaging technique performed by a special catheter with an ultrasound probe attached to the end to achieve cross-sectional imaging. IVUS has a higher sensitivity (90%) and specificity (100%) for the detection of CAC than CAG [92]. However, since ultrasound does not penetrate calcium deposits, the thickness and volume of calcifications cannot be calculated [93]. The high price of the test prevents this technology from being widely promoted.

Optical coherence tomography (OCT) is a new high-resolution cross-sectional imaging mode using light waves whose wavelength is similar to infrared. It has high sensitivity and specificity [94]. Compared to IVUS, it also has a high resolution. OCT is able to calculate the volume of calcification when light penetrates calcium to determine the thickness of the surface calcification [95].

### 6.2. Noninvasive Imaging Testing

Advances in the spatial and temporal resolution of CT have enabled the evaluation of vascular calcification lesions. CT represents the most commonly used noninvasive tool to detect and quantify calcification. The Agatston score, the standard CAC scoring method, classifies the severity of CAC. Many studies conducted in the past decade have also determined the prognostic value of CAC in mortality and overall cardiovascular events in asymptomatic patients, which is now reflected in the main cardiology guidelines [96]. However, in patients with diabetes, CAC and carotid artery intima-media thickness (CIMT) both increase [97]. Studies have also shown that in the entire MESA cohort, CAC scores are more effective than CIMT for predicting cardiovascular events [98]. Traditionally, nonzero calcium scores have been stratified into low (1–100), moderate (101–400), and severe (>400), and a stepwise increase in risk for each category has been noted [99]. A comprehensive meta-analysis tested the prognostic significance of CAC score = 0 in 29,312 individuals who were followed for 50 months and found that the risk of individuals with CAC score = 0 was reduced by 85% compared with individuals with CAC score > 0. An interesting subgroup is the stratum with minimal but nonzero CAC scores (CAC score 1–10). Compared with subjects without CAC on CT, this group experienced a 3-fold increase in cardiac events [100, 101]. Another large review of 44,052 patients who received a CAC score found no clinical risk factors with a CAC score ≥ 400. The event rate of patients was significantly higher than that of subjects with ≥3 risk factors but a CAC score of 0 [102]. In patients with type 2 diabetes, a coronary calcium score greater than 10 predicts cardiovascular events, all-cause mortality, or both and independently predicts cardiovascular events with high sensitivity but low specificity. As a tool for the early evaluation of asymptomatic arterial calcification, it not only is useful as a risk stratification tool for future cerebrovascular events in cardiovascular prevention programs but also provides prognostic information for asymptomatic people.

In recent years, positron emission tomography (PET) has been used for vascular imaging [103]. ^18^-NaF, the imaging agent used for PET, can be located in specific arterial calcifications. Compared to CT, PET detects microcalcifications better [104]. However, due to the high price, the examination is often not considered first when screening for asymptomatic patients for arterial calcification.

Measurement of the IMT has proven to be a powerful indicator of subclinical atherosclerosis and future cardiovascular risk in patients with and without T2DM. The carotid arterial IMT is a well-established surrogate marker for cardiovascular disease. Ultrasonic measurement of the IMT has the advantages of noninvasiveness, good repeatability, and simple operation and is widely used in clinical practice. Several studies have shown that the IMT predicts future myocardial infarction and cerebrovascular disease [105].

### 6.3. Serological Indicators to Assess Severity

Some serological indicators play an important role in the occurrence and development of diabetic vascular calcification. The detection of these indicators plays a key role in the early diagnosis and severity evaluation of complications of diabetic vascular calcification. von Scholten and colleagues [106] found that tumor necrosis factor-*α* is a powerful determinant of cardiovascular disease in patients with type 2 diabetes and is independently associated with vascular calcification mortality. OPG is a member of the tumor necrosis factor (TNF) receptor family implicated in the bone turnover process, osteoporosis, and premature vessel calcification. A recent study identified OPG as an important regulatory molecule in vascular diseases, including cerebral atherosclerosis, and showed its contribution to vessel calcifications in patients with T2DM, suggesting its possible role in the progression of vessel calcification lesions of other vascular beds, such as lower extremity PAD, in patients with diabetes [107]. hsCRP is one of the most sensitive biomarkers of inflammation that induces the release of HMGB-1. In patients with and without T2DM, elevated serum HMGB-1 levels are related to coronary heart disease and are closely related to the severity of coronary artery stenosis [108]. Omentin is an adipokine with anti-inflammatory effects. Yoo et al. and Liu and colleagues [109, 110] performed a cross-sectional study and showed that patients with T2DM and metabolic syndrome, particularly those with carotid artery calcification, exhibited lower omentin-1 levels than healthy subjects. Additionally, Biscetti and colleagues [111] analyzed serum omentin-1 levels in 600 patients with T2DM and found decreased serum omentin-1 levels in patients with T2DM presenting peripheral vascular disease (PAD), and omentin-1 levels are related to disease severity. IL-33/ST2L signaling exerts a cardioprotective effect and protects the myocardium from hypertrophy and myocardial fibrosis after pressure overload. Sst2, the soluble form of suppression of tumorigenicity 2, blocks the binding of interleukin 33 (IL-33) to its transmembrane receptor and the subsequent cardioprotective cascade. Cardellini and colleagues [112] observed significantly increased plasma Sst2 levels in patients with type 2 diabetes. When Sst2 levels increased by one standard deviation, the risk of cardiovascular death was 1.050 (95% CI: 1.006–1.097; *P* = 0.025), suggesting a role for Sst2 in the deterioration of cardiovascular function in high-risk groups. Haptoglobin (Hp) regulates the toxicity of hemoglobin in vitro. Simpson and colleagues found that the HP genotype may help predict the rate of progression of coronary atherosclerosis in patients with type 1 diabetes [113]. This result is consistent with previous studies by Roguin et al., Suleiman et al., and other teams [114, 115].

In previous studies, circulating cardiac biomarkers, including troponin T (TnT) and N-terminal probrain natriuretic peptide (NTproBNP), were considered predictors of cardiovascular adverse events in the general population and patients with diabetes [116]. Recently, in a 13-year follow-up of 8402 participants without cardiovascular disease, the multivariate Cox proportional hazards model revealed that a troponin T level ≥ 14 ng/L (HR: 1.96 (95% confidence interval: 1.57–2.46)) and NTproBNP level > 125 pg/mL (HR: 1.61 (95% confidence interval: 1.29–1.99)) were independent predictors of cardiovascular events [117]. This finding helps distinguish individuals with a high diabetes risk from those with a low diabetes risk and provides incremental risk prediction in addition to commonly used risk markers.

Diabetic foot infection (DFI) is a severe complication of diabetic foot ulcers (DFUs) that dramatically increases the risk of limb amputation and mortality [118]. Many indicators have been identified to determine the severity of DFI and DFU, but relatively few studies are available on their relationship with the prognosis. Procalcitonin (PCT) is a peptide precursor of the hormone calcitonin, which is often undetectable or present at very low concentrations (<0.05 ng/mL) in healthy individuals. Furthermore, PCT is a prognostic marker of severity linked to mortality rates associated with infectious processes [119]. Meloni et al. [120] found that patients who were positive for PCT at admission exhibited a worse prognosis in terms of amputation and mortality than those with normal values. PCT is considered a predictor of death in patients with CLI and hospitalized patients with moderate to severe infections. Regardless of the clinical severity of the infection, its prognostic role should be considered when evaluating these patients.

### 6.4. Glycated Hemoglobin

The UKPDS study found that higher baseline HbA1c levels predicted coronary artery disease in patients with T2DM [121]. For every 1% increase in the glycosylated hemoglobin level, the incidence of cardiovascular events is estimated to increase by 11% to 16% [122]. Epidemiological evidence shows that for every 1% increase in HbA1c levels, cardiovascular events increase by 11%-16%, the incidence of PAD increases by 20%, and the rates of mortality, microvascular complications, and amputation also increase [123]. Anand and colleagues [124] used a multivariate model and found that baseline CAC and HbA1c levels > 7% were powerful predictors of the progression of CAC in patients with type 2 diabetes. Flammer et al. showed that the percentage of circulating OCN+ monocytes increased significantly and the number of OCN^+^ EPCs increased in patients with elevated HbA1c levels compared with those with normal HbA1c levels, indicating a correlation between the osteoblastic drift of EPCs and prediabetes HbA1c levels [125].

### 6.5. Ankle-Brachial Index

The ankle-brachial index (ABI) is critical for the diagnosis of cardiovascular complications of diabetes. The normal range of ABI is 1.00 to 1.40, and a higher prevalence of PAD and its values are all related to an increased risk of cardiovascular events [88]. A low ABI value has a strong predictive value for the occurrence of cardiovascular events and cardiovascular diseases and the overall mortality rate [126]. However, a high ABI value has a stronger correlation with stroke (HR: 2.69) [127]. Additionally, the ABI measurement is also commonly used to diagnose PAD. International guidelines addressing the diagnosis, treatment, and overall management of patients with PAD suggest the ABI as an initial diagnostic test for PAD. However, the ABI evaluation has a low sensitivity for detecting the initial stages of PAD and may not be applicable in patients with diabetes because of calcification of the arterial walls that potentially increases vessel stiffness.

## 7. Treatment and Outcomes of Vascular Calcification in Patients with Diabetes

### 7.1. Blood Sugar Control

Metformin, thiazolidinedione (TZD), sulfonylurea, and insulin are traditionally used to treat diabetes, and their benefits for patients with VC and CVD have also been documented in clinical studies in recent years. As a first-line hypoglycemic drug used in patients with type 2 diabetes, the hypoglycemic effect of metformin is mainly derived from increasing the activity of adenosine monophosphate-activated protein kinase (AMPK) in the liver and reducing gluconeogenesis and adipogenesis. Its vascular protective effect is primarily achieved by inhibiting RANKL, increasing the activity of endothelial nitric oxide synthase (eNOS), and alleviating ROS-related damage [128–130]. In a DIACART cross-sectional cohort study of 198 patients with T2DM, the CAC score of patients treated with metformin was significantly lower than that of patients without metformin treatment [131]. Similarly, 2029 patients with prediabetes had a low CAC score after 14 years of metformin treatment [132]. TZDS exerts its hypoglycemic and anti-inflammatory effects primarily by activating nuclear peroxisome proliferator-activated receptor *γ* (PPAR*γ*). In a rat model induced by *β*-glycerophosphate, pioglitazone, a PPAR*γ* receptor agonist, reduced VSMC calcification through the Wnt pathway [133]. Additionally, studies have shown that pioglitazone reverses tumor necrosis factor-*α*-induced endothelial dysfunction in patients with diabetes [134]. Sulfonylureas play a role by stimulating islet *β*-cells to release insulin. In diabetic mice, glibenclamide downregulates the levels of IL-10, IL-18, and TGF-*β* mRNAs, which are involved in the development of VC [135]. However, the exact mechanism by which sulfonylureas are involved in regulating VC is unclear, and further research is warranted.

Additionally, new hypoglycemic drugs, such as SGLT-2 inhibitors and GLP-1 receptor agonists, have been widely discussed because of their beneficial effects on cardiovascular diseases. SGLT-2 inhibitor is a recently developed ADDS that inhibits glucose reuptake in the proximal tubules of the nephron [136]. SGLT-2 inhibitors not only control blood glucose levels but also control blood pressure, improve renal function, and inhibit proinflammatory cytokines to inhibit calcification [137]. GLP-1 receptor agonists control blood sugar levels by promoting insulin production and inhibiting the release of glucagon. GLP-1 receptor agonists improve inflammation, endothelial function, and myocardial ischemia and lower blood pressure, which are the key protective factors for VC [138]. A comparative review of 20 studies also confirmed these findings [139]. However, to date, no clinical evidence has shown that SGLT-2 inhibitors and GLGLP1 agonists directly affect VC. Thus, further studies are required to improve our understanding of their roles in regulating VC.

### 7.2. Statins

Research by Son and colleagues showed that statins inhibit cellular apoptosis, thereby inhibiting vascular calcification [140]. However, some studies have reached different conclusions: statins help reduce LDL levels, preventing the development of flow-limiting lesions and reducing inflammation. However, their effects on preventing atherosclerosis and calcification are negligible [141].

### 7.3. OPG

The inactivation of osteoprotegerin (OPG) accelerates the progression of calcification [142], and OPG inhibits the differentiation and maturation of osteoclasts (OCs). Treatment with recombinant OPG prevents the occurrence of osteoporosis and vascular calcification and reverses osteoporosis [143]. Currently, continuous research on the mechanism of OPG is identifying its potential as a new target to treat vascular calcification.

### 7.4. Diet

Diabetes is a known risk factor for CAC. A high-sugar diet can induce calcification of human VSMCs by increasing the expression of bone formation markers [144]. In an animal experiment, Zhou and colleagues found that starch or sugar increases the incidence of CV calcification in mice [145]. Another study showed that a high-protein diet reduces renal calcification [146], while low protein intake leads to more severe renal calcification [147].

Unlike traditional methods, reduced calcium intake also causes increased renal calcification and aortic calcification, although high calcium intake is typically not related to vascular calcification in healthy people [148, 149]. Like calcium, phosphate is essential for cell signaling and energy storage in the form of ATP; thus, its concentration must be strictly controlled in the blood. Animal studies have shown a positive correlation between phosphorus intake and aortic and renal calcification [150], but studies on the effects of dietary phosphorus have shown no relationship with CAC [151]. In contrast, higher magnesium intake exerts a more obvious protective effect on CAC. Magnesium is a natural calcium channel blocker that plays an important role in CV. An in vitro study showed that increasing the concentration of magnesium reduces calcification of VSMCs [152]. Additionally, Kang and colleagues performed animal studies and found that high vitamin D intake leads to CV calcification [153], but other studies have indicated that a diet lacking vitamin D leads to an increase in calcified lesions [154]. The relationship between vitamin D disorder and MVC (internally measured vascular calcification) is U-shaped, indicating that excessive or insufficient vitamin D will increase MVC [155].

Oxidative damage is the primary cause of vascular calcification. In patients with diabetes, CV is particularly common, primarily due to increased ROS and lipid oxidation products, such as malondialdehyde. A cross-sectional study showed that the risk of CAC in people who were supplemented with vitamin E (*α*-tocopherol) was significantly higher than that in those who did not receive the supplement [156]. The combination of vitamin C (ascorbic acid) and vitamin E (*α*-tocopherol) reduces the induced calcification of VSMCs [157]. No study has reported a clear association between vitamin A and vascular calcification. Additionally, according to a retrospective analysis, in most studies, the increase in homocysteine levels was significantly positively correlated with coronary artery calcification [158]. Vitamin B12, vitamin B6, and folic acid reduce sulfur-containing amino acids and homocysteine in the body [159].

Vitamin K indirectly prevents calcification through the *γ*-carboxylation of its dependent protein, calcium-binding *γ*-carboxymethylglutamate (MGP). Many patients with CV calcification may also be taking the vitamin K antagonist warfarin, which blocks the *γ*-carboxylation of MGP and further increases calcification [160]. In the only human trial to study cardiovascular calcification, supplementation of 500 *μ*g of phylloquinone daily in the elderly for three years reduced plasma ucMGP levels, significantly slowing the progression of calcification [161].

In summary, regarding diet, patients with a high risk of vascular calcification or extant vascular calcification should avoid eating processed foods rich in trans fatty acids and preservatives. We recommend that the intake of calcium, magnesium, vitamin B, vitamin D, vitamin K, and vitamin C be increased, particularly in patients with diabetes, along with reduced intake of excessive carbohydrates and increased consumption of protein.

## 8. Conclusions

Due to its high incidence and various complications, diabetes decreases the quality of life of patients, causing disability and even death. Among these complications, vascular calcification is one of the most important causes of arteriosclerosis and promotes various macrovascular complications. Currently, diabetic vascular calcification is difficult to completely reverse and eliminate; thus, the prevention of its occurrence and development is very important. Therefore, controllable risk factors should be evaluated in a timely manner, controlled, and corrected, and earlier preventive treatment with a higher cost-benefit ratio should be implemented. Additionally, differences were found in the incidence and prognosis among various vascular beds and in different ethnic groups, but the reasons for these differences remain unclear. Targeted detection and treatment of vascular calcification in various vascular beds and various ethnic groups are the top priority for ameliorating vascular calcification in patients with diabetes.

## Figures and Tables

**Table 1:** International studies on prevalence of vascular calcification.

Reference	Author	Sample size	Ethnic	Calcification site	Conclusion
[76]	Akira Fujiyoshi et al.	1899	Caucasian men *n* = 1067 Japanese men *n* = 832	Coronary artery calcification	Caucasian men in the United States had a higher burden of coronary atherosclerosis than Japanese men, but the ethnic difference was smaller in younger age groups.

[74]	Diane E. Bild et al.	6814	Whites *n* = 2619 Blacks *n* = 1898 Hispanics *n* = 1494 Chinese *n* = 803	Coronary artery calcification	After adjusting the relevant factors, whites have most coronary calcifications, followed by Chinese and Hispanics, and blacks have the least calcifications.

[66]	Matthew J. Budoff et al.	16,560	Asians *n* = 1336 African Americans *n* = 610 Hispanics *n* = 1256	Coronary artery calcification	Increasing prevalence of calcification is noted for all ethnicities with increasing ages. Men had greater prevalence of calcification than women for each ethnicity. Among men, Caucasians are most likely to develop severe CAC (CS > 400), while African Americans are the least likely to develop. But among women, African Americans are the most prone to severe CAC.

[78]	Erbel R et al.	5346	Americans *n* = 2220 Germans *n* = 3126	Coronary artery calcification	Coronary artery calcification prevalence was lower in the United States cohort than the German cohort.

[81]	George Youssef et al.	5886	Whites *n* = 2351 Blacks *n* = 1584 Hispanics *n* = 1265 Chinese *n* = 686	Thoracic aortic calcification	Compared with whites, the progression of thoracic aortic calcification in blacks and Hispanics is significantly slower, and this change is not significant among Chinese.

[82]	A. Bellasi et al.	142	Blacks *n* = 81 Whites *n* = 61	Thoracic aortic calcification	Prevalence and severity of calcification of the thoracic aorta were similar among whites and blacks.

[83]	Tulika Jain et al.	6106	Non-Hispanic Caucasian *n* = 528 African American *n* = 761	Coronary artery calcification	There is no significant difference in coronary artery calcification between black and white men, but it is often higher in black women than in white women. At the same time, the death rate of coronary heart disease in blacks is not significantly higher than that of whites.

[71]	Christina L. Wassel et al.	1417	Blacks *n* = 712 Hispanics *n* = 705	Coronary artery calcification	Among African Americans, each SD increase in European ancestry was associated with an 8% (95% CI, 1.02 to 1.15; *P* < 0.01) higher coronary artery calcification prevalence.

[72]	Peter R. Villadsen et al.	963	Caucasians *n* = 420 South Asian *n* = 543	Coronary artery calcification	The per cent noncalcified plaque composition was lower in Caucasians compared with SA. But the difference of Agatston calcium score (*P* < 0.001) was seen in the nondiabetic group, but not in the diabetic group.

[73]	Erqou et al.	776	Blacks and whites	Coronary artery calcification	Blacks had less CAC than whites and 50% lower odds of a significant CAC score compared with whites. After adjusting for CVD risk factors, this difference still exists.

HNR = Heinz Nixdorf Recall study; MESA = multiethnic study of atherosclerosis; SESSA = Shiga Epidemiological Study of Subclinical Atherosclerosis; SA = South Asian; CAC = coronary artery calcification; CVD = cardiovascular disease; SD = standard deviation.

## Data Availability

The data used to support the findings of this study are available from the corresponding author upon request.
